# Perforated Corneal Ulcer Arising From Gonococcal Keratoconjunctivitis: A Report of Three Cases

**DOI:** 10.7759/cureus.82150

**Published:** 2025-04-12

**Authors:** Nurul Husna Azmi, Rohanah Alias, Valarmathy Vaiyavari, Hannie Ch'ng, Rona Asnida Nasaruddin

**Affiliations:** 1 Ophthalmology, Kuala Lumpur Hospital, Kuala Lumpur, MYS; 2 Ophthalmology, Hospital Canselor Tuanku Muhriz UKM (Universiti Kebangsaan Malaysia), Kuala Lumpur, MYS

**Keywords:** adult gonococcal keratoconjunctivitis, gonococcal keratoconjunctivitis, neisseria gonorrhoeae, ophthalmia neonatorum, peripheral ulcerative keratitis

## Abstract

This report aims to raise awareness among ophthalmologists about gonococcal keratitis, which may present with peripheral corneal thinning and ulceration accompanied by mucopurulent discharge. A high index of suspicion facilitates early diagnosis, timely intervention, and improved visual outcomes. We describe three cases: a 49-year-old male presenting with right eye redness, vision loss, and purulent discharge from both the eye and urethra, whose examination revealed peripheral corneal thinning with perforation and iris plugging; a 28-year-old healthy male with a two-day history of decreased visual acuity, photophobia, and purulent discharge in the left eye, with findings of a corneal perforation at the 10 o’clock position and peripheral thinning; and a 19-year-old male reporting seven days of right eye redness, purulent discharge, lid swelling, and vision loss, preceded by a five-day history of urethral discharge and dysuria, with examination showing peripheral corneal thinning and ulceration with corneal perforation. Corneal swabs from all three patients tested positive for *Neisseria gonorrhoeae*, confirming gonococcal keratoconjunctivitis leading to corneal perforation. They were treated with intravenous ceftriaxone (2 g daily for 14 days) and topical antibiotics, and all subsequently underwent corneal transplantation. In conclusion, gonococcal keratoconjunctivitis is a rapidly progressive condition that can result in corneal perforation, endophthalmitis, and blindness, underscoring the critical need for early recognition and prompt ophthalmologic referral.

## Introduction

Gonorrhea, caused by *Neisseria gonorrhoeae*, is a widespread sexually transmitted infection (STI) that can affect multiple mucosal sites, including the conjunctiva. Although ocular involvement is less frequent, it poses a serious risk to vision, particularly when complications such as corneal perforation develop [1]. In adults, transmission to the eye typically occurs through autoinoculation, often via contaminated hands following contact with infected genital secretions [2].

Historically, gonococcal conjunctivitis has been predominantly associated with neonates, acquired during childbirth from an infected mother [3]. However, in recent years, the incidence of gonorrhoeal eye infections in the Eastern countries of Ireland has surged sevenfold, escalating from 6.9 cases per 100,000 individuals in 2003 to 49.2 cases per 100,000 individuals in 2012 [4]. Comparable upward trends have been observed in England [5] and Australia [6]. This trend highlights the need for greater clinical awareness of gonococcal ocular infections beyond the neonatal period.

The condition presents with acute ocular symptoms, including severe conjunctival inflammation, purulent discharge, eyelid swelling, and eye pain. Without prompt intervention, the infection can rapidly progress to corneal ulceration and perforation, given *N. gonorrhoeae*'s ability to invade intact corneal epithelium. Due to its aggressive nature, early diagnosis and immediate initiation of appropriate systemic and topical antibiotic therapy are essential to prevent permanent vision loss [7,8].

One of the major diagnostic challenges is distinguishing gonococcal keratitis from peripheral ulcerative keratitis (PUK), an immune-mediated condition commonly associated with systemic autoimmune diseases such as rheumatoid arthritis and granulomatosis with polyangiitis [9].

This report highlights the importance of considering gonococcal keratitis in patients presenting with peripheral corneal ulceration resembling PUK, particularly in sexually active individuals or those with concomitant mucopurulent conjunctivitis. Increasing awareness of this entity among ophthalmologists can facilitate early recognition and timely intervention, ultimately improving patient outcomes.

## Case presentation

Case 1

A previously healthy 49-year-old male presented with redness and reduced vision in the right eye, accompanied by purulent discharge over the past 10 days. Notably, urethral discharge with dysuria had been experienced three days before the onset of eye symptoms. The patient, who was married and had two children, reported a positive history of multiple sexual partners in the past three months. Seeking medical attention on the third day of illness, he was initially diagnosed with bacterial conjunctivitis and treated with topical antibiotics by a general practitioner. However, after one week of treatment, the right eye vision deteriorated, leading to a referral to the ophthalmology department.

**Figure 1:**
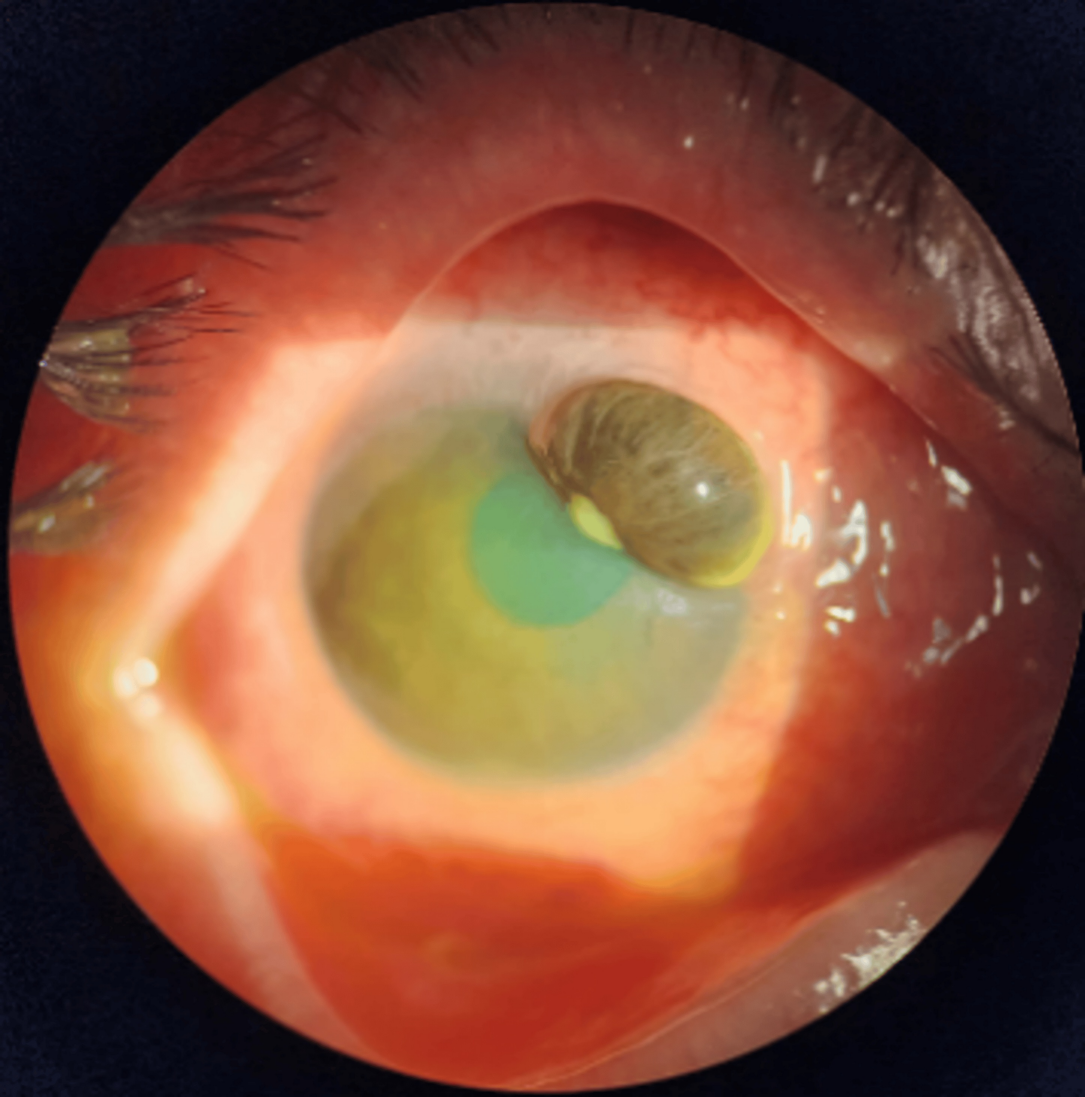
Right eye showing peripheral cornea thinning with perforation and iris plugging

Upon assessment by the ophthalmologist, the patient exhibited counting-finger visual acuity in the right eye and 6/6 in the left eye. The right eyelid was oedematous with injected conjunctiva and purulent discharge. Examination revealed cornea perforation with iris plugging at the peripheral superonasal area, along with mild anterior chamber activity, but no hypopyon or fibrin (Figure 1). Bright Scan ultrasonography (B-scan) indicated a flat retina with clear vitreous. The left eye examination was normal.

**Figure 2:**
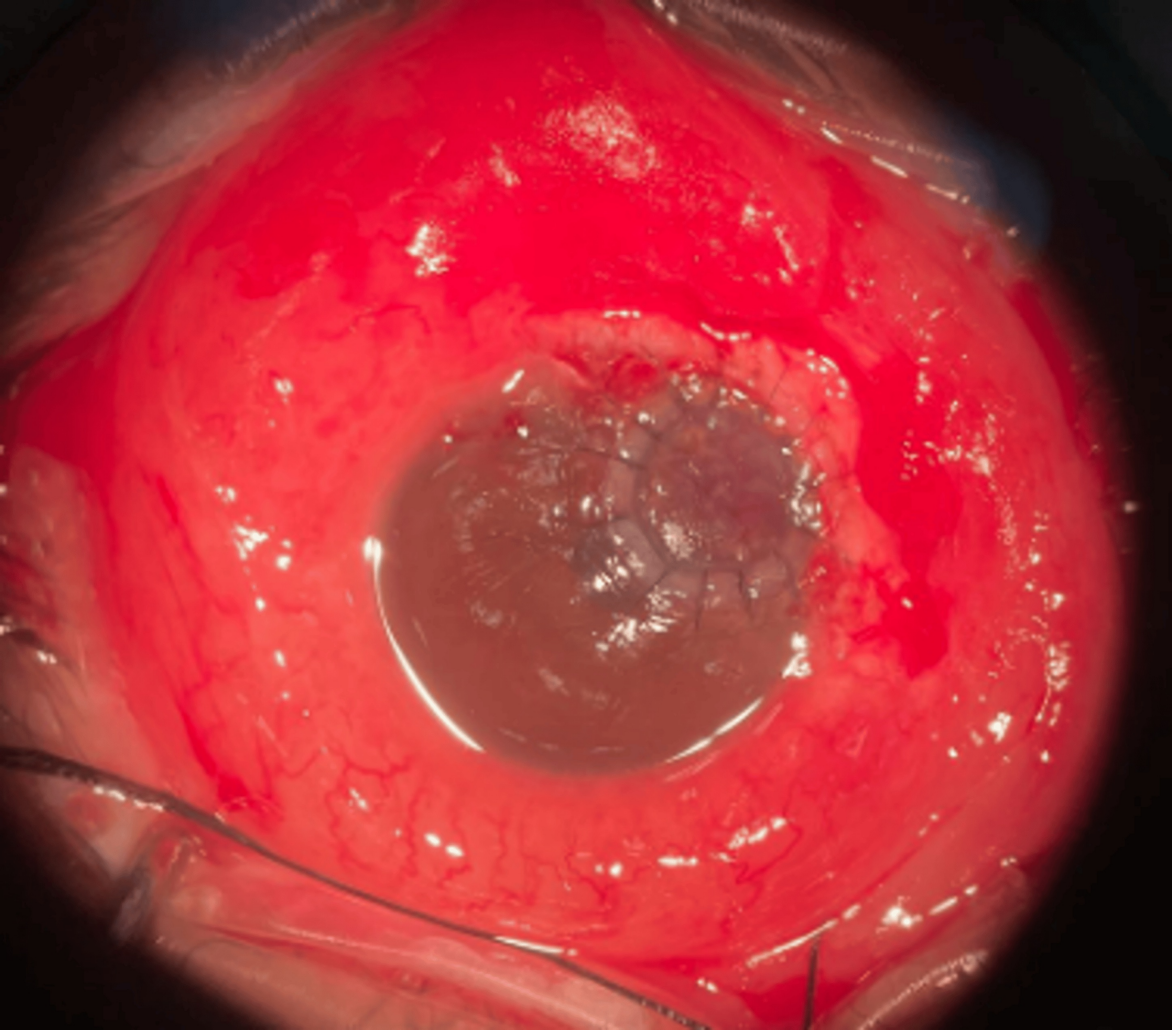
Right eye, post penetrating keratoplasty

Gram stain of the eye discharge did not reveal any organisms, but a positive urine culture for *N. gonorrhoeae* and *Chlamydia trachomatis*, sensitive to ceftriaxone, confirmed the diagnosis of right eye corneal perforation secondary to gonococcal keratoconjunctivitis. The patient underwent treatment with intravenous ceftriaxone (2 g daily for 14 days), oral doxycycline (100 mg twice daily for seven days), and topical levofloxacin 0.5% hourly over the right eye. A temporary tarsorrhaphy was performed, and the case was referred to the cornea team, and a full-thickness cornea graft was performed on the right eye (Figure 2). Six months post-operation, the patient's vision was 6/30, with no signs of graft rejection, and the patient was not on any topical antiglaucoma medication.

Case 2

 A 28-year-old man with no known medical illness presented with a sudden onset of decreased visual acuity, photophobia, and purulent discharge in his left eye over the course of two days. Notably, he had an episode of ocular trauma when a part of processed chicken accidentally fell into his left eye while working in a chicken processing factory before the onset of eye symptoms. The patient denied any history of sexual promiscuity and reported no other systemic complaints.

**Figure 3:**
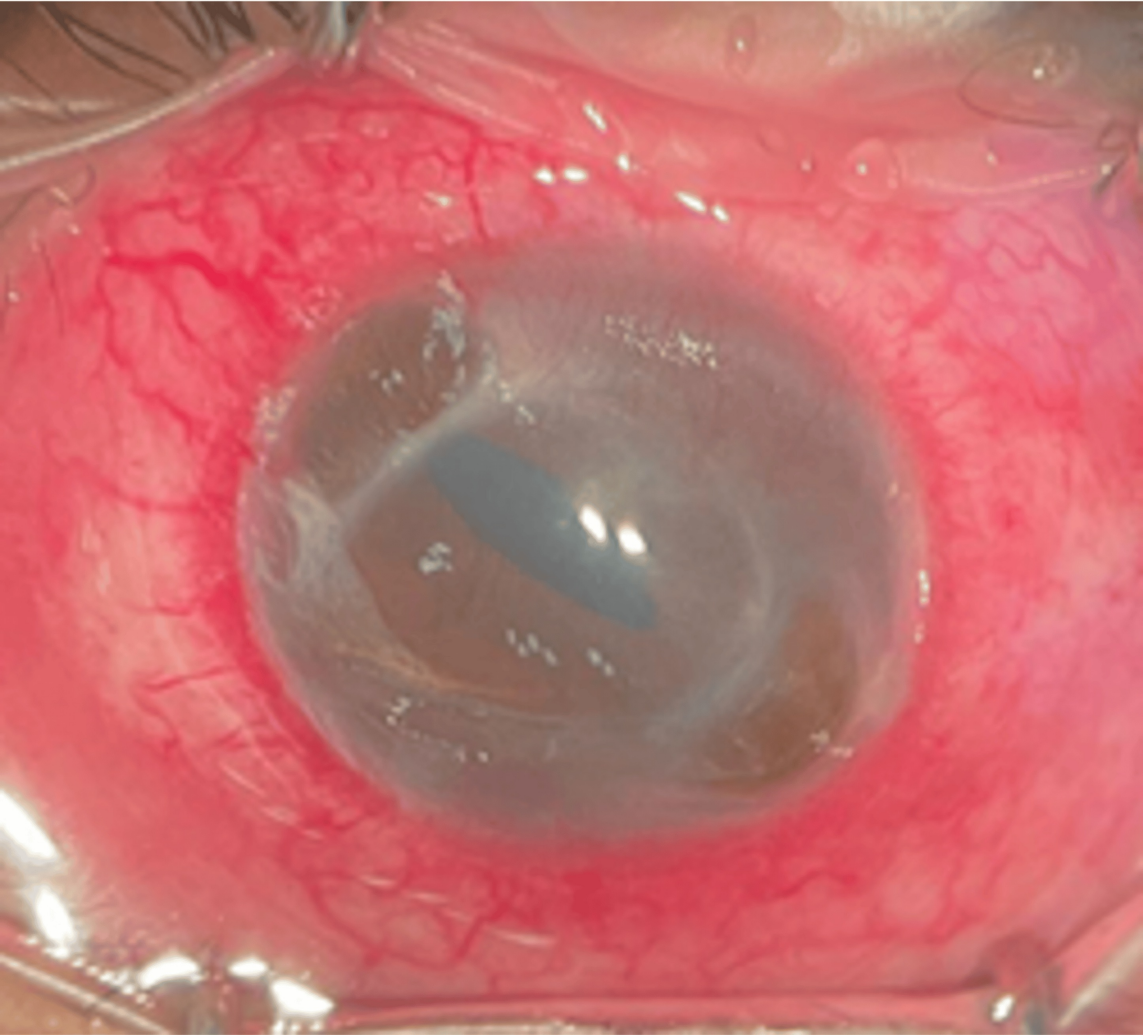
Left eye showing 360 degrees cornea thinning with perforation at 10 o’clock and descemetocele at 5 o’clock.

Upon arrival at the ophthalmology department, his visual acuity was 6/6 in the right eye but reduced to counting fingers in the left eye. Slit lamp examination revealed an injected conjunctiva in the left eye with purulent discharge. Further examination unveiled a corneal perforation at the peripheral 10 o’clock area, characterized by evidence of iris prolapse. Additionally, there was an area of cornea thinning with descemetocele at the peripheral five o’clock. Mild anterior chamber activity was noted, without hypopyon or fibrin (Figure 3). B-scan results indicated a flat retina with clear vitreous. The right eye examination was unremarkable.

Gram stain analysis of the purulent discharge from the left eye revealed gram-negative diplococci, and the culture from the ocular discharge tested positive for *N. gonorrhoeae*, confirming the diagnosis of left eye cornea perforation secondary to gonococcal keratoconjunctivitis.

**Figure 4:**
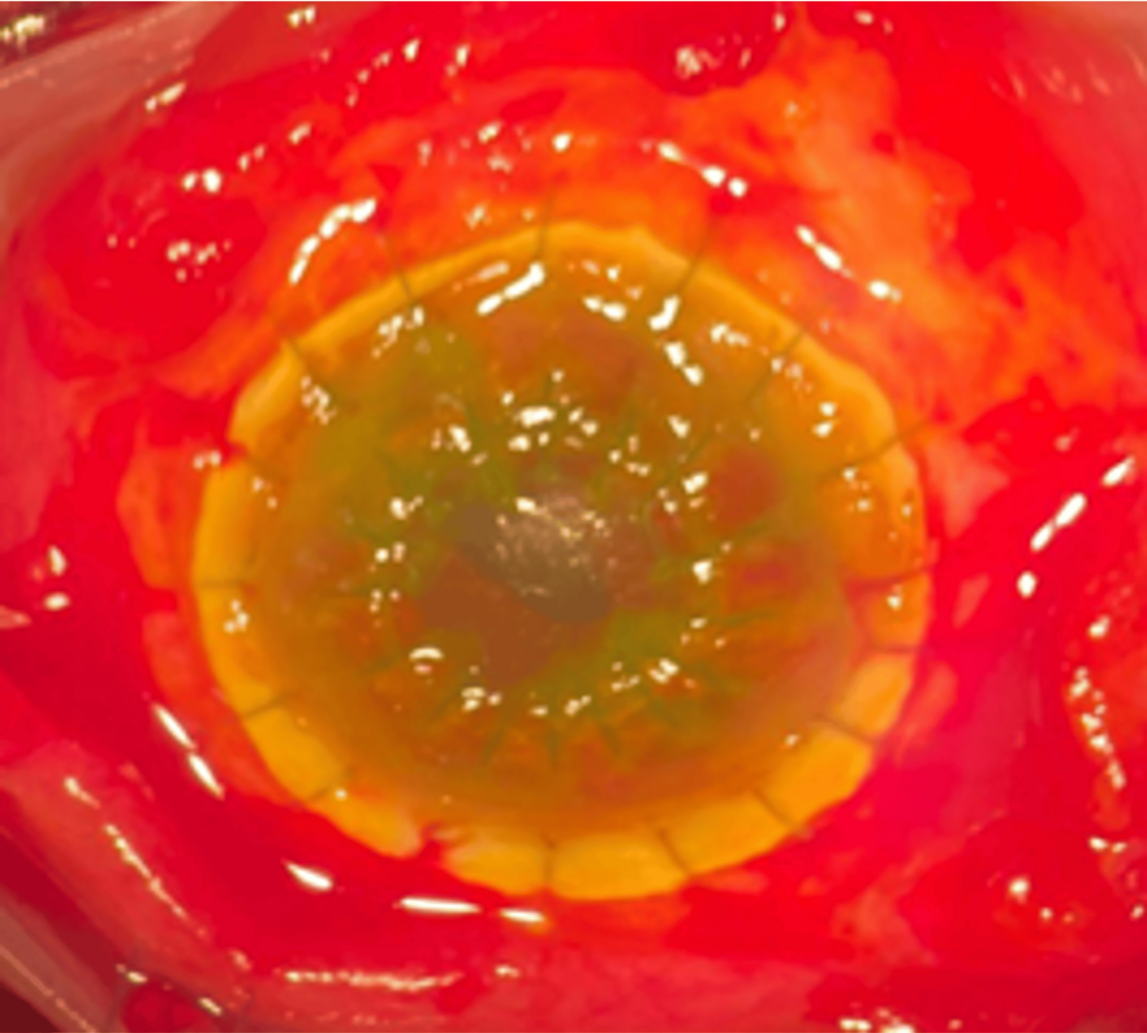
Doughnut shape lamellar keratoplasty

The patient was promptly initiated on intravenous ceftriaxone (2 g daily for 14 days) and received topical moxifloxacin 0.5% hourly over the left eye. A temporary tarsorrhaphy was performed, and the case was referred to the cornea team, where the patient underwent a left eye lamellar keratoplasty (Figure 4). Two months post operation, the patient's vision remained at counting fingers, with no signs of graft rejection, and the patient was on two topical antiglaucoma medications. As the patient was from Indonesia, subsequent follow-up was conducted in that country.

Case 3

A sexually active 19-year-old man presented with redness in the right eye persisting for seven days. He reported associated symptoms of purulent discharge, lid swelling, and reduced vision. Preceding the onset of eye symptoms, he complained of urethral discharge with dysuria for the past five days.

**Figure 5:**
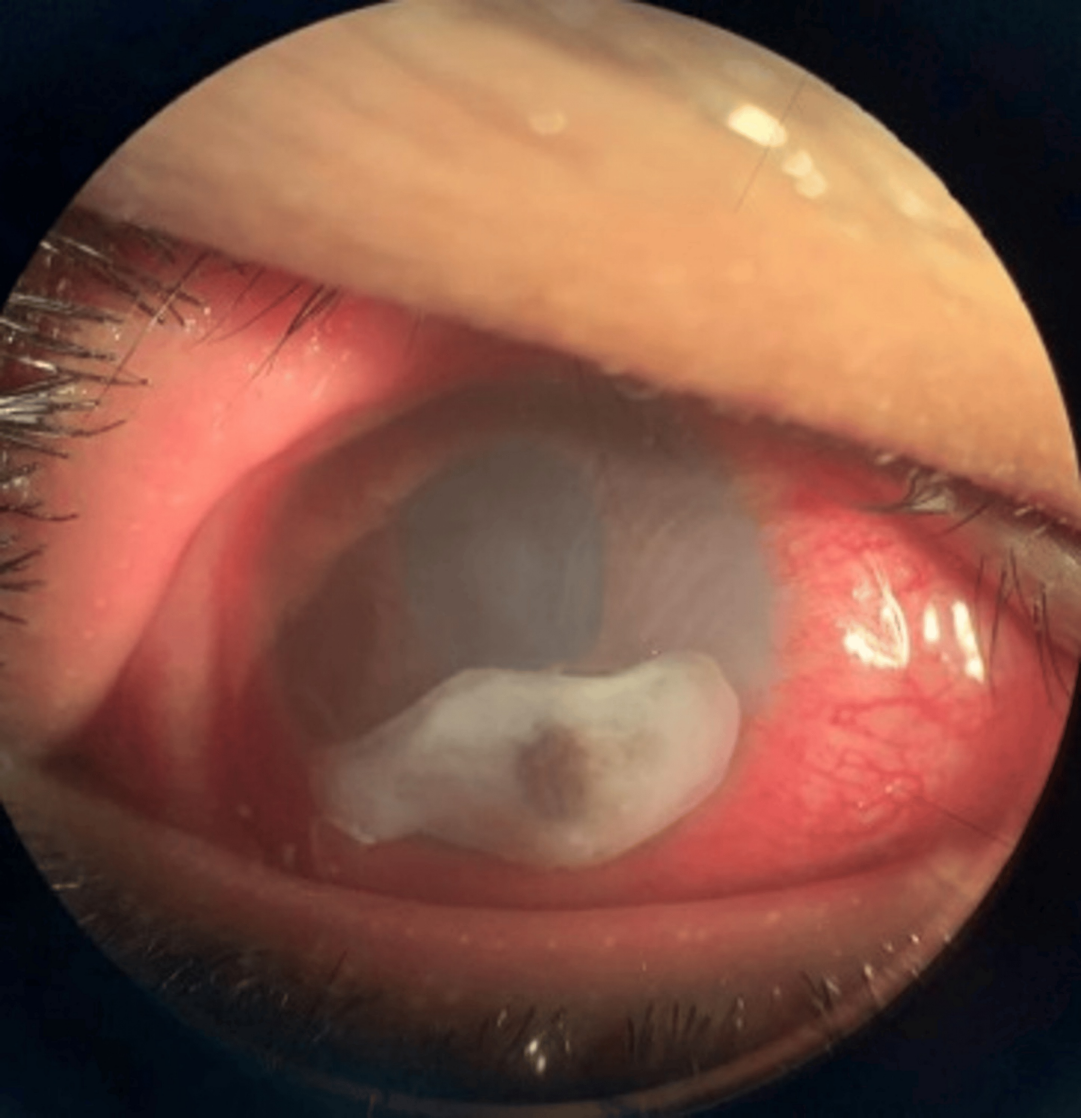
Right eye inferior cornea ulcer with iris plugging

Examination revealed counting-fingers visual acuity in the right eye, accompanied by mucopurulent discharge and lid oedema. The conjunctiva appeared injected, and examination of the cornea indicated thinning from five o’clock to two o’clock, with infiltration inferiorly from five o’clock to seven o’clock. Iris plugging was observed inferiorly, along with a shallow anterior chamber (Figure 5). Despite these findings, the Seidel test was negative, suggesting a self-sealed perforated cornea ulcer. B-scan imaging showed no vitreous loculation, and the examination of the left eye was unremarkable.

Both the corneal swab and the urine culture tested positive for *N. gonorrhoeae*, identified as kidney-shaped gram-negative diplococci, confirming the diagnosis of a self-sealed cornea perforation in the right eye secondary to gonococcal keratoconjunctivitis. The patient was fitted with a bandage contact lens while awaiting a corneal donor and received treatment with topical antibiotics (levofloxacin 0.5% hourly and ceftazidime 5% hourly) and intravenous ceftriaxone (2 g daily for 14 days).

**Figure 6:**
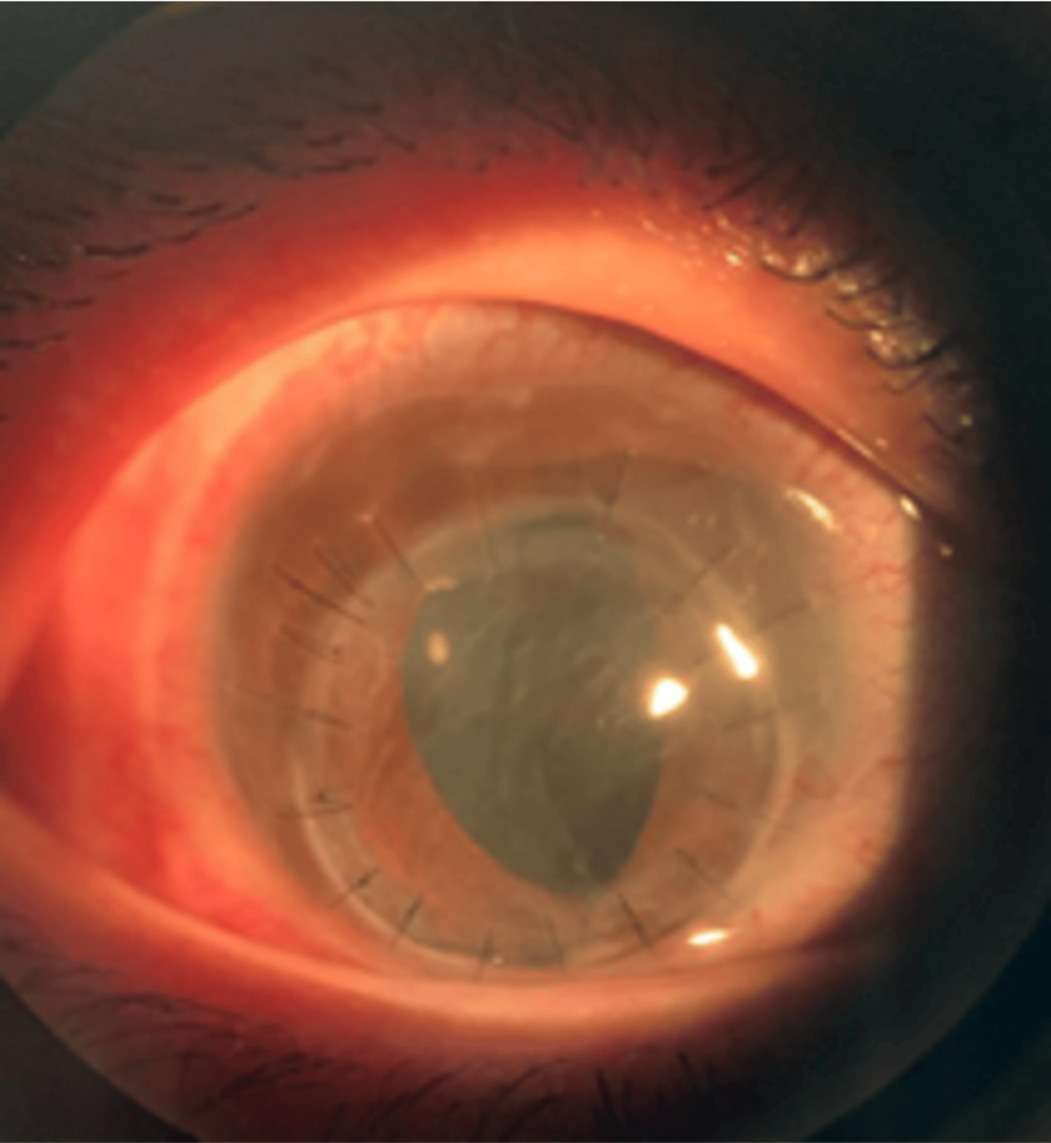
Right eye post tectonic keratoplasty

A tectonic penetrating keratoplasty was performed on the right eye, and follow-up assessments over six months revealed an improvement in visual acuity from counting fingers to 6/24 (Figure 6) with no signs of graft rejection.

 Table 1 gives a summary of the three cases.

**Table 1:** Summary of the three cases

Case	Age/Gender	History	Ocular findings	Confirmatory Test	Treatment	Outcome Post Corneal Transplantation
1	49-year-old male	Right eye redness, vision loss, and purulent discharge from both the eye and urethra for 10 days, with positive history of sexual promiscuity.	Peripheral superonasal corneal thinning with perforation and iris plugging.	Eye swab: No growth Urine culture & sensitivity: *Neisseria gonorrhoea*, *Chlamydia trachomatis*	IV ceftriaxone 2g daily for 14 days, Oral doxycycline 100mg twice daily for seven days, topical antibiotics, corneal transplantation	Preoperative vision : Counting Fingers; Six months postoperative vision : 6/30; Signs of graft rejection : Nil
2	28-year-old male	Left eye reduced vision, photophobia, and purulent discharge for two days. Patient denied history of sexual promiscuity.	Peripheral corneal thinning and corneal perforation at 10 o’clock position	Eye swab : *Neisseria gonorrhoea*	IV ceftriaxone 2g daily for 14 days, topical antibiotics, corneal transplantation	Preoperative vision : Counting Fingers; Two months postoperative vision : Counting Fingers (Subsequent follow-up was conducted in Indonesia); Signs of graft rejection : Nil
3	19-year-old male	Right eye redness, purulent discharge, lid swelling, and vision loss, preceded by a history of urethral discharge and dysuria for seven days, with positive history of sexual promiscuity.	Peripheral corneal thinning and ulceration with corneal perforation	Eye swab : *Neisseria gonorrhoea* Urine culture & sensitivity : *Neisseria gonorrhoea*	IV ceftriaxone 2g daily for 14 days, topical antibiotics, corneal transplantation	Preoperative vision : Counting Fingers; Six months postoperative vision : 6/30; Signs of graft rejection : Nil

## Discussion

Gonococcal conjunctivitis was thought of as a disease in neonates previously; however, recently, it has also become an increasing issue in other age groups [10,3]. Our aim in this report is to highlight different manifestations of gonococcal conjunctivitis. Gonococcal conjunctivitis is an ophthalmic infection caused by *N. gonorrhoeae*, a gram-negative diplococcus. It is a potentially sight-threatening condition due to the unique capability of the bacteria to penetrate both intact corneal and conjunctival epithelium [7]. In adults, the infection is transmitted through contact between the eyes and infected genital secretions from individuals with genital gonorrhoea whereas in neonates, transmission typically occurs during delivery, exposing them to infectious vaginal secretions [11].

Cases of adult gonococcal conjunctivitis are rare [11]; however, a recent study in Ireland estimated a prevalence of 0.19 cases per 1000 patients evaluated for eye emergencies [3]; the majority is seen in young adult males, with recent gonorrhoea outbreak in adult predominantly amongst men who have sex with men (MSM) and young heterosexual populations [1-3,8].

Neonatal infection is usually accompanied by a history of suspected or confirmed maternal gonorrhoea infection. Bacterial conjunctivitis can occur at any time, but gonococcal conjunctivitis needs to be considered in symptomatic neonates after the first day of life, specifically, days two to five. Common presentations in neonates include conjunctival injection and chemosis, eyelid oedema, mucopurulent discharge, and preauricular lymphadenopathy [11].

However, the presentation in adults may vary, making the diagnosis of gonococcal conjunctivitis challenging. The clinical course typically includes acute pain, conjunctival injection, chemosis, and a profuse purulent discharge. Other than the usual presentation, Perez et al. reported a case of gonococcal infection presenting as a rapidly progressing corneal ulcer [7]. Another case series of gonococcal infection reported that lid involvement was a common presentation and may resemble preseptal cellulitis [3]. Belga et al. reported a case of gonococcal infection in which the patient presented with keratitis and a corneal epithelial defect in one eye, and conjunctivitis with purulent discharge in the other eye [12]. In the present report, all patients initially showed thinning of the peripheral cornea, which eventually led to perforation, resembling the features of peripheral ulcerative keratitis. However, as highlighted by Hassanpour et al., gonorrhoea infection stands out among infectious culprits, posing a rare yet serious threat to the cornea as it can precipitate peripheral ulcerative keratitis, ultimately leading to corneal perforation [13]. 

One of the major diagnostic challenges is distinguishing gonococcal keratitis from PUK, an immune-mediated condition commonly associated with systemic autoimmune diseases such as rheumatoid arthritis and granulomatosis with polyangiitis, as in our cases. Clinically, both conditions can present with peripheral corneal ulceration, thinning, and stromal infiltration, leading to misdiagnosis and delays in appropriate treatment. However, unlike PUK, gonococcal keratitis typically features copious mucopurulent discharge, rapid progression to corneal perforation, and a marked inflammatory response, with a higher likelihood of anterior chamber involvement [13].

Given its potential for rapid deterioration and severe visual consequences, early identification of gonococcal keratitis is critical. A detailed history, including sexual activity and possible urogenital symptoms, is essential for raising clinical suspicion. Laboratory confirmation with Gram stain, culture, and polymerase chain reaction (PCR) aids in distinguishing gonococcal keratitis from immune-mediated causes. Prompt treatment with systemic ceftriaxone and intensive topical antibiotics is necessary to prevent corneal perforation and endophthalmitis [1-3,7,8,11-14]. Alternative antibiotics for treating urogenital *N. gonorrhoeae* include a single oral dose of cefixime 800 mg in combination with a single oral dose of azithromycin 1 g [15]. All sexual partners should be tested and treated, even if asymptomatic, to prevent reinfection [15].

## Conclusions

Early identification of gonococcal keratitis is crucial, as the infection progresses rapidly and can lead to severe complications, including endophthalmitis and blindness, making prompt ophthalmologic referral and immediate treatment essential for preserving vision.

## References

[1] Kirkcaldy RD, Weston E, Segurado AC, Hughes G (2019). Epidemiology of gonorrhoea: a global perspective. Sex Health.

[2] Bastion ML, Prakash K, Siow YC, Loh SS (2006). Bilateral corneal perforation in a sexually active adult male with gonococcal conjunctivitis. Med J Malaysia.

[3] McAnena L, Knowles SJ, Curry A, Cassidy L (2015). Prevalence of gonococcal conjunctivitis in adults and neonates. Eye (Lond).

[4] Fitzgerald M, Cooney F, Ennis O, Downes P, Clarke S (2013). Gonorrhoea - a major public health challenge in Dublin, Wicklow and Kildare. Epi-Insight.

[5] Savage EJ, Marsh K, Duffell S, Ison CA, Zaman A, Hughes G (2012). Rapid increase in gonorrhoea and syphilis diagnoses in England in 2011. Euro Surveill.

[6] Lahra MM (2013). Gonococcal surveillance Australia: quarter 3, 2012. Commun Dis Intell Q Rep.

[7] Pérez CE, Bravo JS, Espinal A (2016). A 30-year-old male with corneal opacity and a rapidly progressing ulcer. Infectio.

[8] Lessing JN, Slingsby TJ, Betz M (2019). Hyperacute gonococcal keratoconjunctivitis. J Gen Intern Med.

[9] Gomes BF, Santhiago MR (2021). Biology of peripheral ulcerative keratitis. Exp Eye Res.

[10] Tan AK (2019). Ophthalmia neonatorum. N Engl J Med.

[11] Belga S, Gratrix J, Smyczek P, Bertholet L, Read R, Roelofs K, Singh AE (2019). Gonococcal conjunctivitis in adults: case report and retrospective review of cases in Alberta, Canada, 2000-2016. Sex Transm Dis.

[12] Hassanpour K, H ElSheikh R, Arabi A (2022). Peripheral ulcerative keratitis: a review. J Ophthalmic Vis Res.

[13] Eslami M, Benito-Pascual B, Goolam S, Trinh T, Moloney G (2022). Case report: use of amniotic membrane for tectonic repair of peripheral ulcerative keratitis with corneal perforation. Front Med (Lausanne).

[14] Unemo M (2015). Current and future antimicrobial treatment of gonorrhoea - the rapidly evolving Neisseria gonorrhoeae continues to challenge. BMC Infect Dis.

[15] Lee JS, Choi HY, Lee JE, Lee SH, Oum BS (2002). Gonococcal keratoconjunctivitis in adults. Eye (Lond).

